# Other PHAC publications

**DOI:** 10.24095/hpcdp.44.4.07

**Published:** 2024-04

**Researchers from the Public Health Agency of Canada also contribute to work published in other journals and books. Look for the following articles published in 2023 and 2024:**

Bouchouar E, Levine MJ, Ileka-Priouzeau S, Dave S, et al. Exploring challenges and opportunities in detecting emerging drug trends: a socio-technical analysis of the Canadian context. Can J Public Health. 2023. https://doi.org/10.17269/s41997-023-00842-w

Dun-Dery F, Xie J, Winston K, Salvadori MI, et al. Post-COVID-19 condition in children 6 and 12 months after infection. JAMA Netw Open. 2023;6(12). https://doi.org/10.1001/jamanetworkopen.2023.49613

Frehlich L, Turin TC, Doyle-Baker P, Lang JJ, et al. Mediation analysis of the associations between neighbourhood walkability and greenness, accelerometer-measured physical activity, and health-related fitness in urban dwelling Canadians. Prev Med. 2024;178: 107792. https://doi.org/10.1016/j.ypmed.2023.107792

Leatherdale ST, Amores A, Blanger RE, […] Jiang Y. Youth perception of difficulty accessing cannabis following cannabis legalization and during the early and ongoing stages of the COVID-19 pandemic: repeat cross-sectional and longitudinal data from the COMPASS study. Arch Public Health. 2023;81(1):214. https://doi.org/10.1186/s13690-023-01224-x

Palis H, Haywood B, McDougall J, […] Burgess H, et al. Factors associated with obtaining prescribed safer supply among people accessing harm reduction services: findings from a cross-sectional survey. Harm Reduct J. 2024;21(1):5. https://doi.org/10.1186/s12954-024-00928-9

Sayfi S, Charide R, Elliott SA, […] Stevens A, et al. A multimethods randomized trial found that plain language versions improved adults understanding of health recommendations. J Clin Epidemiol. 2024;165:111219. https://doi.org/10.1016/j.jclinepi.2023.11.009

Shaver N, Beck A, Bennett A, […] Garritty C, Subnath M, […] Traversy G, Graham E, et al. Screening for hypertension in adults: protocol for evidence reviews to inform a Canadian Task Force on Preventive Health Care guideline update. Syst Rev. 2024;13(1):17. https://doi.org/10.1186/s13643-023-02392-1

Toigo S, Katzman DK, Vyver E, McFaull SR, Iynkkaran I, Thompson W. Eating disorder hospitalizations among children and youth in Canada from 2010 to 2022: a population-based surveillance study using administrative data. J Eat Disord. 2024;12(1):3. https://doi.org/10.1186/s40337-023-00957-y

Warkentin MT, Ruan Y, Ellison LF, […] Demers AA, […] Brenner DR. Progress in cancer control leads to a substantial number of cancer deaths avoided in Canada. JNCI Cancer Spectr. 2023;7(6):pkad105. https://doi.org/10.1093/jncics/pkad105

Zhu F, Zhao Y, Arnold DL, […] Bonner C, Graham M, […] Knox N, […] Van Domselaar G, et al. A cross-sectional study of MRI features and the gut microbiome in pediatric-onset multiple sclerosis. Ann Clin Transl Neurol. 2023. https://doi.org/10.1002/acn3.51970

Ziam S, Lanoue S, McSween-Cadieux E, […] Jean E, et al. A scoping review of theories, models and frameworks used or proposed to evaluate knowledge mobilization strategies. Health Res Policy Syst. 2024;22(1):8. https://doi.org/10.1186/s12961-023-01090-7

